# Adriamycin does not damage podocytes of zebrafish larvae

**DOI:** 10.1371/journal.pone.0242436

**Published:** 2020-11-13

**Authors:** Maximilian Schindler, Antje Blumenthal, Marcus Johannes Moeller, Karlhans Endlich, Nicole Endlich

**Affiliations:** 1 Department of Anatomy and Cell Biology, University Medicine Greifswald, Greifswald, Germany; 2 Department of Nephrology and Clinical Immunology, RWTH Aachen University Clinic, Aachen, Germany; Karpagam Academy of Higher Education, INDIA

## Abstract

Podocytes are highly specialized epithelial cells that are essential for an intact glomerular filtration barrier in the kidney. Several glomerular diseases like focal segmental glomerulosclerosis (FSGS) are initially due to podocyte injury and loss. Since causative treatments for FSGS are not available until today, drug screening is of great relevance. In order to test a high number of drugs, FSGS needs to be reliably induced in a suitable animal model. The zebrafish larva is an ideal model for kidney research due to the vast amount of offsprings, the rapid development of a simple kidney and a remarkable homology to the mammalian glomerulus. Zebrafish larvae possess a size-selective glomerular filtration barrier at 4 days post fertilization including podocytes with interdigitating foot processes that are connected by a slit membrane. Adriamycin is an anthracycline which is often used in mice and rats to induce a FSGS-like phenotype. In this study, we aimed to induce a similar phenotype to zebrafish larvae by adding adriamycin to the tank water in different concentrations. Surprisingly, zebrafish larvae did not develop glomerular injury and displayed an intact filtration barrier after treatment with adriamycin. This was shown by (immuno-) histology, our filtration assay, *in vivo* imaging by 2-photon microcopy, RT-(q)PCR as well as transmission electron microscopy. To summarize, adriamycin is unable to induce a podocyte-related damage in zebrafish larvae and therefore major effort must be made to establish FSGS in zebrafish larvae to identify effective drugs by screenings.

## Introduction

Chronic kidney disease (CDK) with a global prevalence of 9.1% and an increase by 29.3% since 1990 is a major burden for both patients and public healthcare [1]. Within CKD, Glomerulopathies are the main causes for the development of nephrotic syndromes worldwide [2]. Especially, focal segmental glomerulosclerosis (FSGS) is a severe glomerular pathohistological condition which often results in end stage renal disease [3]. FSGS is strongly associated with podocyte damage, hypertrophy and a loss of these post-mitotic cells [4]. Activated parietal epithelial cells, accumulation of extracellular matrix and finally glomerular scarring follow podocyte injury [5]. Since regenerative mechanisms of podocytes are poorly understood, the renal filtration barrier is permanently affected. Until now, no causative drugs or therapies are available underlining the urgent need of screening methods that identify drugs for glomerulopathies.

In the past, it became clear that the zebrafish larva is an excellent model to study kidney development, function and morphology [6, 7]. Today, it is apparent that the zebrafish could be an ideal model for drug screening due to a large number of advantages compared to mice and rats. The quick development of hundreds of *ex utero* fertilized eggs per week, a simple kidney (pronephros) as well as transparency open many possibilities in that field. The zebrafish larva develops a filtrating pronephros approximately 40 hours post fertilization (hpf) which is composed of only one single glomerulus connected to two tubules [8]. Moreover, the morphology of the zebrafish glomerulus shows high similarities to that of mammals. Additionally, the interdigitating podocytes of the zebrafish larvae express slit membrane specific proteins like nephrin and podocin that are the prerequisite for a size selectivity of the filtration barrier. Thus, the knockdown of nephrin or podocin by specific Morpholinos resulted in a disrupted filtration barrier and a proteinuric phenotype in zebrafish larvae [9]. The renal filtration of zebrafish larvae can even be observed *in vivo* by utilizing a strain that expresses eGFP fused to the vitamin-D binding protein as an indicator for a proper filtration barrier [10].

In order to induce glomerular injury in animal models, adriamycin (ADR or Doxorubicin) is widely used. ADR is an anthracycline that is commonly utilized to induce FSGS in rodents [11–13]. The efficacy of ADR is strongly dependent on the rodent strain it is used on. While being effective on most rat strains, only a few mouse strains are susceptible to ADR nephropathy [14]. Beside this, side effects like cardiomyopathy, a small range for the dose and a high degree of technical administration expertise are disadvantages of this model [15]. Regarding zebrafish, administration of ADR at an early stage to the tank water induced developmental defects of the pronephros [16]. Injection of ADR in 3 dpf old larvae resulted in severe cardiovascular off target effects and was deemed unsuitable as an injury model [17].

The aim of this study was to induce injury to the pronephric glomerulus of zebrafish larvae in order to mimic FSGS and therefore to create a drug screening model. To this end, ADR was administered into the tank water of 7 dpf old larvae. Subsequently, zebrafish larvae were analyzed by RT-(q)PCR, a filtration assay, in histological sections and *in vivo* 2-photon microscopy. In contrast to rodent models, zebrafish larvae did not respond to ADR treatment with a FSGS-like phenotype.

## Materials and methods

### Zebrafish husbandry

Zebrafish were held at standard conditions in tanks with circulating water at 27°C and and a light cycle of 14:10 h (14 light: 10 dark). Mating was induced in small groups in mating tanks overnight and eggs were collected in the morning. Larvae were raised in E3 medium at 28.5°C, as previously described [18]. In order to track podocytes, the ET strain (Tg(-35.1wt1a:eGFP); mitfa^w2/w2^; mpv17^a9/a9^, kindly provided by Dr. C. Englert, Jena, Germany) was used for all experiments except for the filtration assay [18, 19]. This was conducted with DBP larvae (Tg(-3.5fabp10a:gc-eGFP), which was kindly provided by Dr. B. Anand-Apte, Cleveland, USA [20]. Larvae were fed twice a day from 6 to 9 dpf with GEMMA Micro 75 (Zebcare, Neederwert, Netherlands). Anesthesia for *in vivo* imaging and euthanasia was conducted with tricaine (Sigma-Aldrich, St. Louis, Missouri, USA).

All prerequisites of the German animal protection law were met and experiments were performed in accordance with the guidelines of the federal agencies in Mecklenburg-Western Pomerania (LALLF M-V). The responsible ethics committee within the LALLF M-V approved the experiments with zebrafish larvae older than 6 dpf.

### Adriamycin treatment

At 7 dpf the E3 medium was changed to E3 containing 0 (Ctrl), 20, 40, or 60 μM adriamycin (Doxorubicin-HCL, Selleckchem, Houston, Texas, USA). The medium was refreshed at 8 dpf to remove feeding debris. After 48 h adriamycin was washed out three times and larvae were either fixed for histological studies or homogenized in TRI-Reagent (Sigma-Aldrich) for mRNA analysis.

### Filtration assay

DBP larvae expressing eGFP as a fusion protein with the vitamin D-binding protein (~68kDa) were anesthetized with tricaine (0.1–0.5%) at 7 dpf before treatment and the caudal artery was focused with a Leica TCS SP5 10x air objective (Wetzlar, Germany). After treatment with ADR, larvae were again anesthetized and imaged. For each concentration and each round of treatment (4) 10 corresponding larvae were imaged before and after treatment, resulting in 40 larvae per concentration. The vascular eGFP intensity was measured with ImageJ (National Institutes of Health, Bethesda, MD, USA) and the intensity after treatment was normalized to the fluorescence intensity before treatment.

### Histology

Hematoxylin and eosin (HE) staining was performed on plastic sections. Larvae were fixed overnight in 2% PFA at 4°C and dehydrated in an ascending series of ethanol on the next day. Following dehydration, larvae were treated with infiltration medium, embedded in Technovit^®^ 7100 (Kulzer, Hanau, Germany) and hardened overnight. Plastic sections (4 μm) were cut with a rotational microtome (Jung RM2055, Leica Biosystems, Wetzlar, Germany). Sections were stained with Gill´s hematoxylin, alcoholic eosin and mounted with Eukitt^®^ (Sigma-Aldrich). Images were taken with an Olympus BX50 microscope (Tokio, Japan).

### Immunofluorescence

Cryosections (6 μm) were cut on a Microm HM 560 microtome (Thermo Fisher Scientific, Waltham, Massachusetts, USA) and immunofluorescence staining was performed as previously described [21]. Nephrin staining was carried out overnight at 4°C with 1:2000 rabbit anti-zebrafish nephrin (gift of Dr. A. Majumdar, Uppsala, Sweden) and Alexa-Fluor-647 anti-rabbit antibody (Invitrogen, Carlsbad, California, USA). An antigen retrieval step was necessary for podocin staining. Briefly, cryosections were boiled in Tris-HCL pH 9.0 for 10 min. After antigen retrieval and blocking, cryosections were incubated with a rabbit anti-podocin antibody (Proteintech, Rosemont, Illinois, USA; 1:200) o.n. at 4°C followed by an incubation with Alexa-Fluor-647-labeled anti-rabbit antibody (Invitrogen). Nuclei were stained with Hoechst 33342 (Sigma-Aldrich) and sections were finally mounted with Mowiol (Carl Roth, Karlsruhe, Germany). Confocal microscopy of cryosections was carried out on a Leica TCS SP5 with a 40x and a 63x oil immersion objective.

### RNA isolation and cDNA synthesis

RNA of 8–12 larvae was isolated with TRI-Reagent according to manufacturer´s protocol. The QuantiTect reverse transcription kit (Qiagen, Hilden, Germany) was used for cDNA synthesis out of 1 μg RNA.

### RT-PCR and RT-qPCR

PCRs were performed as previously described [21]. In brief, DreamTaq DNA Polymerase (Thermo Fisher Scientific) was used for RT-PCR and RT-qPCR was carried out with iQ SYBR green Supermix (BioRad, Hercules, California, USA) on an iCycler Thermal Cycler (BioRad). Primer sequences were the following: *nphs1*-FOR: CAATGTCCCTAACCCGCACT, *nphs1*-REV: ACGCCTCACATTGCAGAGAA, *nphs2*-FOR: GAAGCAAGACGTCAGGCACA, *nphs2*-REV: GGTATGTTGAGGACCACGGC, *eef1a1l1*-FOR: AAGGAGGGTAATGCTAGCGG, *eef1a1l1*-REV: GGGCGAAGGTCACAACCATA. Controls without cDNA template, without RNA and samples without reverse transcriptase (RT) in the RT reaction were included in every run. Melting curves for each primer were also checked.

### Ultrastructural analysis

Larvae were fixed with 4% glutaraldehyde, 1% PFA and 1% sucrose in 0.1 M HEPES at 4°C overnight. Dehydration in an ascending ethanol series was performed after post-fixation in 2% osmium tetroxide. The larvae were embedded in EPON 812 (Serva, Heidelberg, Germany) and cut with an Ultracut UCT ultramicrotome (Leica, Wetzlar, Germany). Ultrathin sections (70 μm) were drawn on a copper grip and contrasted with Sato´s lead stain and 5% uranyl acetate for 5 min. Images were acquired with a LIBRA 120 transmission electron microscope (Zeiss, Oberkochen, Germany).

### *In vivo* imaging by 2-photon microscopy

*ET* larvae were embedded, positioned in 0.6% low melting agarose and anesthetized as described earlier [22]. Before treatment and 48 h post treatment with 0, 20, 40 and 60 μM ADR automated z-stacks over a distance of up to 90 μm with 1 μm distance between frames were acquired with a LSM710MP (Carl Zeiss Microimaging, Jena, Germany) and a Chameleon Ti-Sapphire Laser (Chameleon, Coherent, Santa Clara, CA, USA). A 20x (1.0 NA) water immersion objective was used with an excitation wavelength of 890 nm for eGFP. Movies were generated with ImageJ.

### Statistics

Mann-Whitney *U* test was used for analysis in which p values <0.05 were considered statistically significant. Error bars represent ±SD. All experiments were repeated four times with 18 to 58 larvae per treatment group in each round. A total of 784 larvae were used for this study.

## Results

### ADR treatment did not induce edema formation

**Fig 1:**
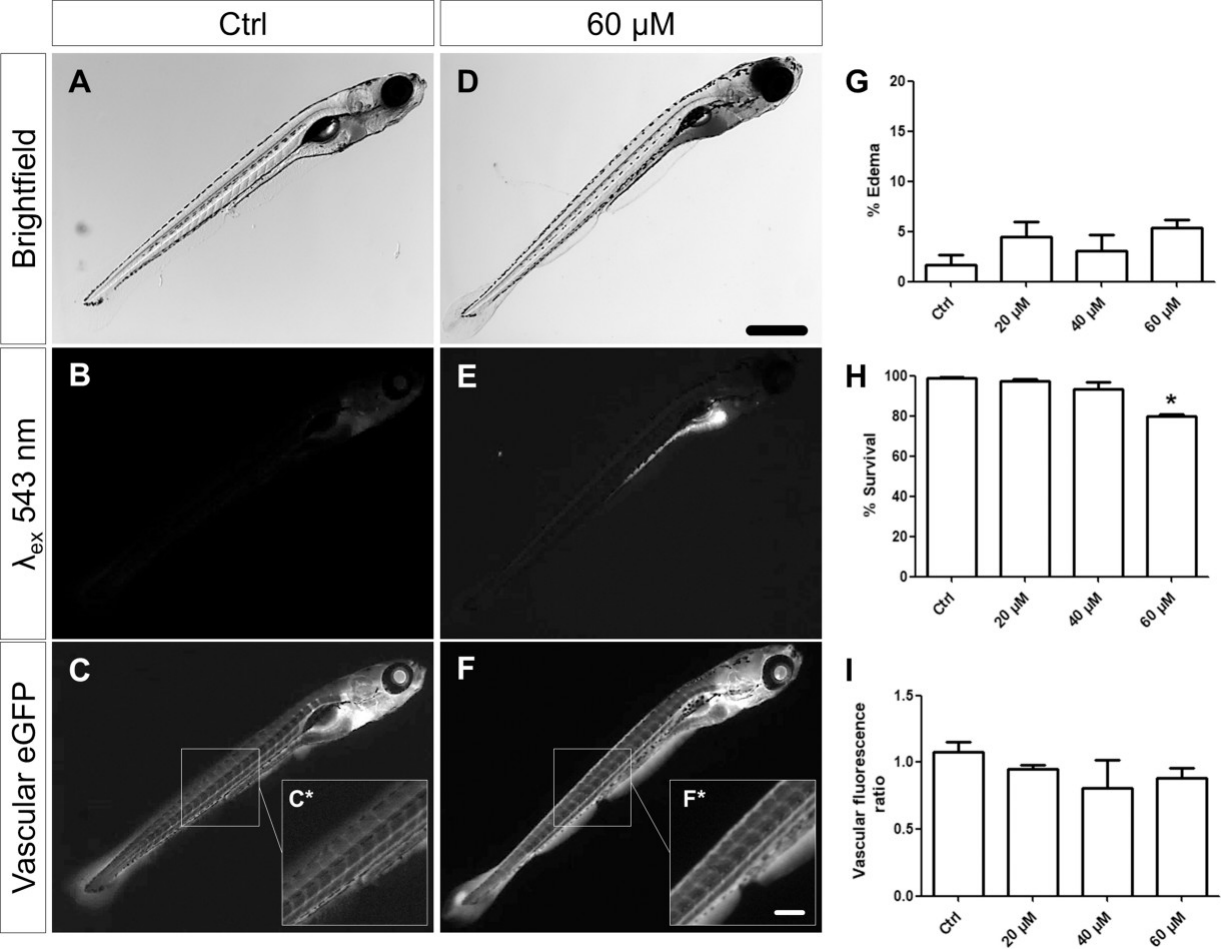
Evaluation of phenotype, autofluorescence, vascular eGFP fluorescence, survival and edema rate of larvae after treatment. Treated larvae did not develop periocular or pericardial edemas at a significant rate (**A, D, G**). However, high concentrations of ADR caused an increase in mortality of the larvae (**H**). All doses of ADR led to a strong accumulation of ADR in the intestinal tract which caused a remarkable autofluorescence over a wide spectrum (**B, E**). Quantification of the vascular eGFP fluorescence of DBP larvae (Tg(-3.5fabp10a:gc-eGFP) after ADR treatment did not reveal a significant vascular reduction of the eGFP-DBP fusion protein (~68 kDa) **(C, F, I)**. Scale bar in **D** represents to 200 μm, in F* to 50 μm. *P = 0,03.

Zebrafish larvae were treated with three different concentrations of ADR (20, 40 and 60 μM) in tank water for 48 h. Theses doses were chosen on the basis of Zennaro at al. [16] and preliminary experiments. The mortality of zebrafish larvae increased after the treatment with 60 μM ADR but no evident edema formation was observed. However, ADR in lower concentrations did not lead to any edema formation (periocular, pericardial or yolk sac). In contrast to the zebrafish larvae treated with 60 μM ADR, low dose treated larvae survived similar to the larvae of the control group (Fig 1).

### The filtration barrier remains intact during ADR treatment

To study the function of the filtration barrier, a screening zebrafish strain expressing the 68 kDa eGFP-vitamin D-binding protein (Tg(-3.5fabp10a:gc-eGFP)) in the blood was used [20]. Under healthy conditions, all vessels of the larvae show a strong fluorescence of eGFP in contrast to a loss of the fluorescence if the filtration barrier becomes leaky which results in the excretion of the eGFP-vitamin D-binding protein [10].

After the treatment of the zebrafish larvae (7 dpf) with different concentrations of ADR, the fluorescence of the vasculature remained unchanged (Fig 1), indicating an intact filtration barrier. Beside the eGFP fluorescence, a strong red fluorescence in the intestine was observed when exposed to an excitation wavelength of 543 nm which was caused by the uptake of ADR by the larvae (Fig 1).

### ADR did not affect the morphology of the glomeruli

**Fig 2:**
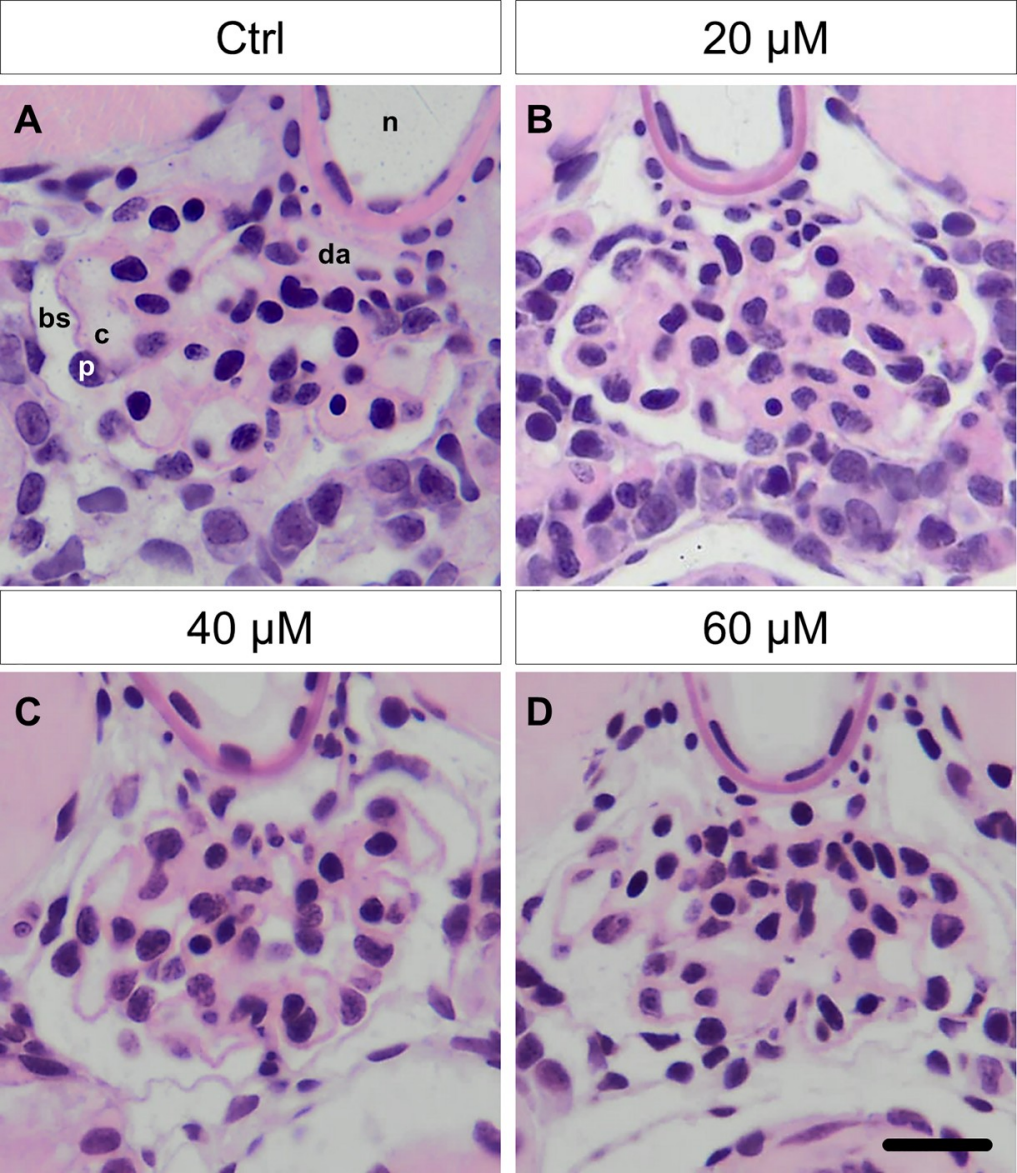
Glomeruli of ADR-treated larvae did not show morphological alterations demonstrated by HE staining on histological sections. A loss of podocytes or a Bowman‘s space edema could not be observed (**A-D**). **n**: notochord, **da**: dorsal aorta, **c**: capillary, **p**:podocyte, **bs**: Bowman‘s space. Scale bar represents 10 μm.

In order to examine the glomerular morphology in ADR-treated zebrafish larvae at 9 dpf, histological sections were cut and were stained with hematoxylin and eosin (HE). We have found that ADR treatment at different concentrations did not change the morphology and the size of the glomerulus, respectively. Furthermore, neither a loss of podocytes nor areas of naked glomerular basement membrane (GBM) were observed suggesting that ADR has no effect on the glomerular structure (Fig 2).

### ADR did not influence the expression of slit membrane proteins

**Fig 3:**
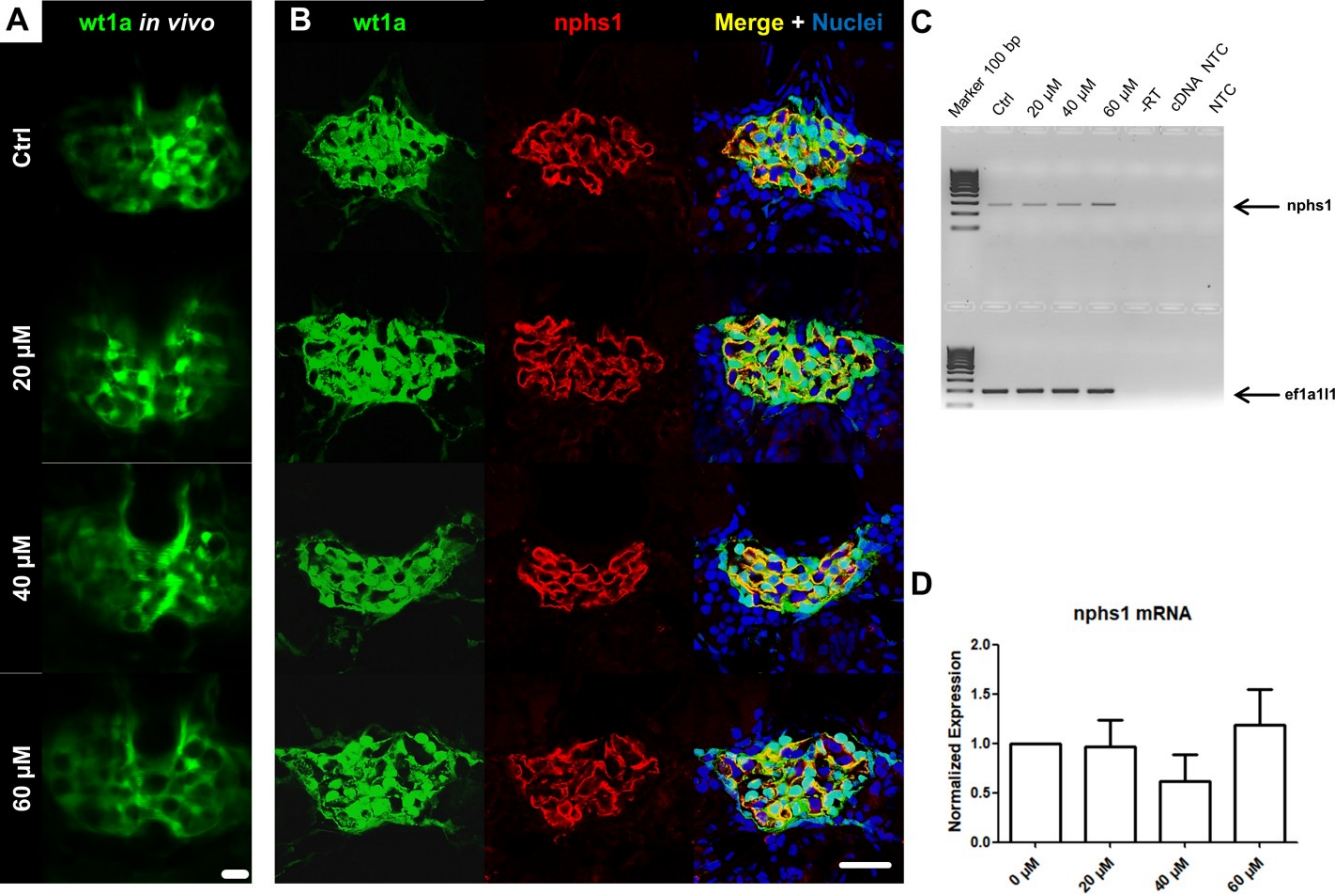
Evaluation of wt1a expression *in vivo* and immunostaining of cryosections for the slit membrane protein nephrin in ADR-treated larvae. 2-PM *in vivo* imaging revealed no morphological changes or glomerular abnormalities in treated larvae **(A)**. Immunostainings showed that ADR treatment neither reduces the expression of the pronephric transcription factor wt1a nor changes the expression pattern of nephrin **(B)**. RT-PCR **(C)** and RT-qPCR **(D)** confirmed that nephrin mRNA expression was not altered in ADR-treated larvae at 9 dpf. Scale bars represent 10 μm.

In order to assess the integrity of the pronephric filtration barrier of zebrafish larvae, the *ET* strain was used which expresses eGFP in podocytes under the control of the wt1a promotor. Cryosections of ADR-treated larvae revealed that endogenous eGFP fluorescence was not diminished in the presence of ADR (Fig 3). In accordance with this, immunostainings of the slit membrane proteins nephrin and podocin showed a well-organized, meandering pattern in the pronephric glomerulus in each group. Furthermore, we have not found a significant regulation of podocin and nephrin mRNA due to ADR treatment quantified by RT-(q)PCR (Fig 3 and S1 Fig). These results hint towards an insusceptibility of larval zebrafish podocytes to ADR.

### ADR had no impact on glomeruli *in vivo*

Living larvae (9 dpf) were imaged and the glomerular integrity was assessed before and after ADR treatment by 2-PM. *In vivo* z-stacks of treated larvae did not show impaired glomeruli and were indistinguishable from controls (Fig 3A and S2-S6 Movies in S1 File).

### ADR has no impact on the ultrastructure of the glomerular filtration barrier

**Fig 4:**
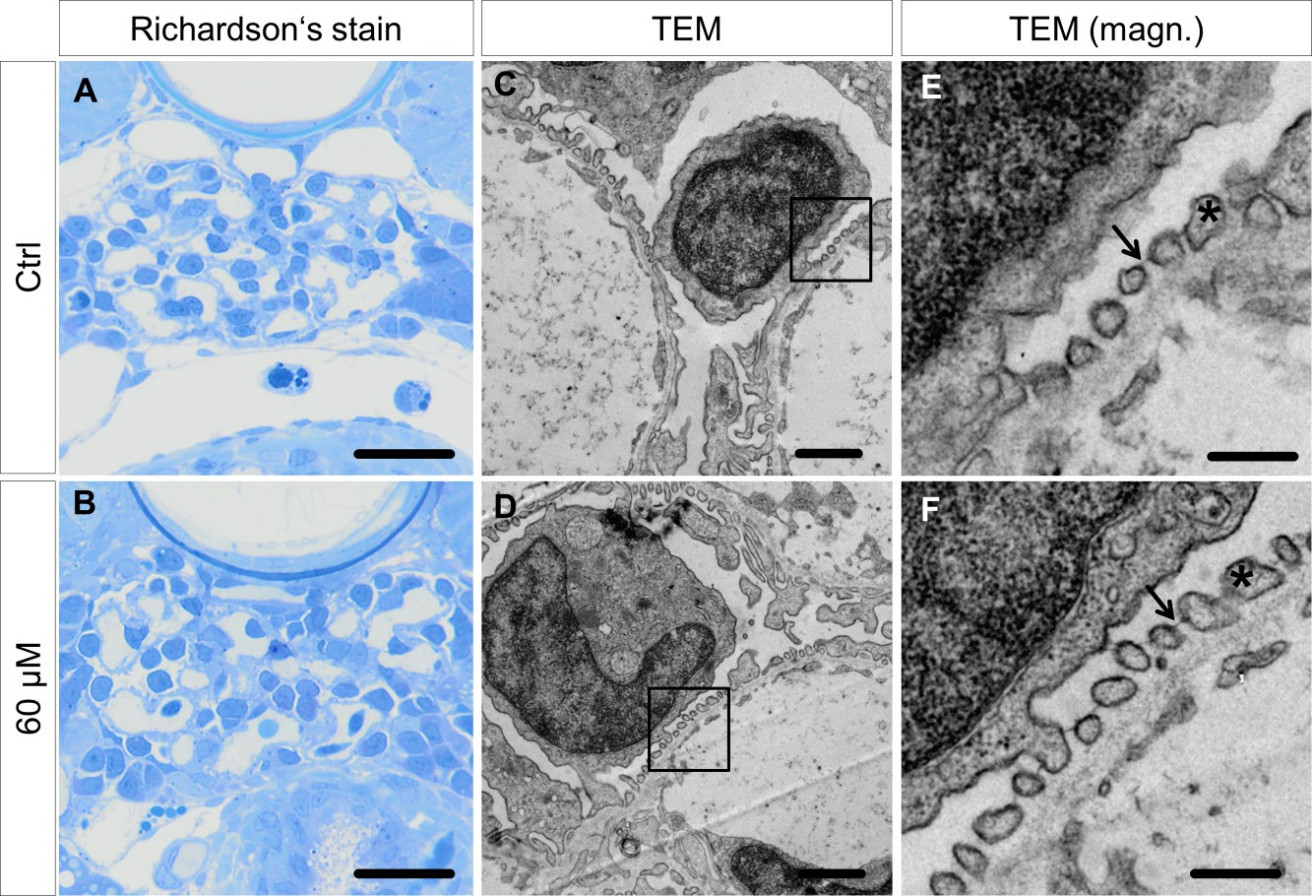
Morphology and ultrastructure of larval glomeruli. Morphological investigations by Richardson‘s staining of semithin sections (500 nm) did not show abnormalities of the glomerular morphology due to ADR treatment (**A, B**). Ultrastructural analysis of the pronephric filtration barrier confirmed the abscence of a glomerular phenotype (**C-F**). The interdigitating pattern of podocyte foot processes (asterisk in **E** and **F**) is present in both control was well as 60 μM ADR-treated larvae. The slit membrane as a highly organized structure connecting foot processes was not affected by ADR (arrows in **E** and **F**). Scale bars in **A** and **B** represent 10 μm, in **C** and **D** 1 μm, in **E** and **F** 200 nm.

The filtration barrier of larval zebrafish is remarkably similar to the mammalian counterpart, especially in terms of morphology. Podocyte foot processes (FP) are basolaterally connected by slit-membrane and adhere to the three-layered GBM on top of a fenestrated endothelium. In many pathologic conditions, the slit-membrane perishes and foot processes flatten and merge. This condition is called effacement. To find out whether FP morphology is affected by ADR administration, ultrathin sections were examined with a transmission electron microscope (TEM). Ctrl- and ADR-treated (60 μM) larvae both showed highly organized and interdigitating foot processes which are connected by the slit membrane. Podocyte foot process effacement was not observed in ADR-treated larvae. Furthermore, the morphology of the glomerular endothelium was unchanged as well as the thickness of the GBM was similar to the Ctrl larvae (Fig 4).

## Discussion

The zebrafish larva is a well-established model organism to investigate pronephric development, morphology and function. Due to the low maintenance costs and rapid maturation of even highly specialized cells like functional podocytes within 3.5 dpf [6], it offers considerable advantages over mammalian organisms. The enormous reproduction rate of several hundred eggs per week makes it an ideal model for high throughput screening of drugs. Since kidney diseases and especially the ones affecting the glomerulus are on an alarming rise, it is of crucial importance to find causative medical treatments. The first step to establish a drug screening assay in zebrafish larvae is induction of a reliable and reproducible glomerular injury that mimics diseases like FSGS.

An induction of glomerular injury in zebrafish larvae has been achieved successfully by several approaches in the past. The injection of morpholinos at early developmental stages which repress mRNA translation of essential proteins for the pronephric development or ones that target podocyte specific mRNAs directly have shown convincing results [18, 23, 24]. Although this technique provides robust results, it is limited to represent developmental defects of the pronephros and no direct damage to fully functional podocytes.

In order to target mRNA translation in the pronephric glomerulus in later developmental stages, Vivo-morpholinos have successfully been used by our group [25]. This technique offers induction of injury at stages in which podocytes are fully functional but is not suitable for high-throughput experiments because of the time-consuming manner in which intravenous injections in zebrafish larvae have to be performed. An excellent method to specifically induce damage to podocytes in zebrafish larvae has been carried out with the nitroreductase-metronidazole system [10, 26]. Podocyte apoptosis, formation of pseudocysts, podocyte detachment and proteinuria can be induced by adding metronidazole to the medium without injection [21, 27]. Although this model is very robust and provides easily reproducible results, it is limited to one zebrafish strain.

Since ADR is commonly used in rodent models to induce glomerular injury, we tested the effect of this compound on glomeruli of zebrafish larvae. It was reported that the application of ADR at early larval stages affects their proper development and the formation of the filtration barrier [16, 28]. Since injections of ADR into the cardiac sinus venosus in zebrafish larvae at 3 dpf causes off-target cardiovascular effects, ADR was being considered unsuitable for induction of an pronephric injury via injection [17].

In the present study, we found that application of ADR to the tank water of zebrafish larvae with a fully functional pronephric filtration barrier caused a significant increase in mortality at 60 μM but did not cause an occurrence of hallmarks for nephrotic syndromes such as pericardial or periorbital edema. In accordance with this, a renal loss of eGFP of DBP larvae could not be observed in treated larvae indicating an intact and size-selective filtration barrier. Interestingly, ADR accumulated in the intestinal tract of treated larvae which led to a strong fluorescence when excited with a wavelength of 543 nm. According to Motlagh and colleagues, ADR has its peak of fluorescence at ~591 nm with an emissive bandwidth of 150 nm [29]. Therefore, zebrafish strains that express endogenous fluorophores in this bandwidth (e.g. mCherry with λ_Em_ ~ 610 nm, RFP with λ_Em_ ~ 588 nm) as well as conjugated antibodies that emit in this bandwidth (e.g. Cy3 with λ_Em_ ~ 570 nm) cannot be used in ADR treatments.

In order to identify possible pre-nephrotic pathohistological alterations of the glomerular filtration barrier, HE staining and immunofluorescence staining for nephrin and podocin of *ET* larval glomeruli were performed. Treated larvae did not show histologic hallmarks of glomerular injury such as podocyte loss, dilated capillaries or Bowman´s space edema. Immunofluorescence staining did not reveal a changed protein expression of nephrin or podocin. RT-(q)PCR results undermined the unaltered expression of these two important podocyte proteins upon ADR treatment.

Since transparent zebrafish larvae are ideal for *in vivo* observations, imaging of living ADR-treated larvae was performed by 2-PM. Z-stacks of glomeruli displayed no injury in living larvae after treatment which strongly corroborates the results of a missing nephrotic phenotype.

Ultrastructural analysis of treated larvae further revealed that ADR did not have any impact on the glomerular filtration barrier. Even larvae that were treated with 60 μM still showed a fenestrated endothelium, a normal GBM and interdigitating foot processed connected by a slit membrane. Foot process effacement or a thickening of the GBM was not induced, which are further hallmarks of glomerular injury.

Most rat strains but only a few mouse strains are susceptible to ADR nephropathy [14]. Zheng and colleagues found that ADR susceptibility in mice is a Mendelian trait. A decreased expression of the Prmt7 protein of the *DOXNPH* locus is responsible for susceptibility to ADR nephropathy in mice [30]. It was shown that zebrafish larvae express prmt7 at early developmental stages [31, 32] but little is known about the expression of prmt7 at later stages.

In conclusion, zebrafish larvae are an excellent animal model to study kidney function *in vivo*. However, ADR is not a suitable drug to induce acute podocyte or glomerular injury in zebrafish larvae. Further substances have to be tested in order to find a drug that induces glomerular injury and mimics FSGS in zebrafish larvae.

## Supporting information

S1 Fig Effects of ADR treatment on the expression of podocin. Similar to nephrin, podocin showed a meandering expression along the slit membrane in glomeruli throughout all ADR concentrations (A). These results were corroborated by podocin mRNA analysis of treated larvae via RT-PCR and RT-qPCR (B, C). Scale bar represents 10 μm.
(TIF)

S1 File S2-S6 Movies. Representative 2-photon microscopy *in vivo* z-stacks of ET larvae before treatment at 7 dpf and after treatment at 9 dpf. No signs of glomerular injury such as podocyte loss or bowman's space edema were seen after ADR treatment. Treated larvae displayed an intact glomerular cytoarchitecture, primary processes of podocytes could be resolved in all groups.
(ZIP)

S1 Raw images (PDF)
