# Supplementary figures and images for: Adriamycin does not damage podocytes of zebrafish larvae
Source: PLoS One. 2020 Nov 13;15(11):e0242436. doi: 10.1371/journal.pone.0242436 (PMC7665694; doi:10.1371/journal.pone.0242436)

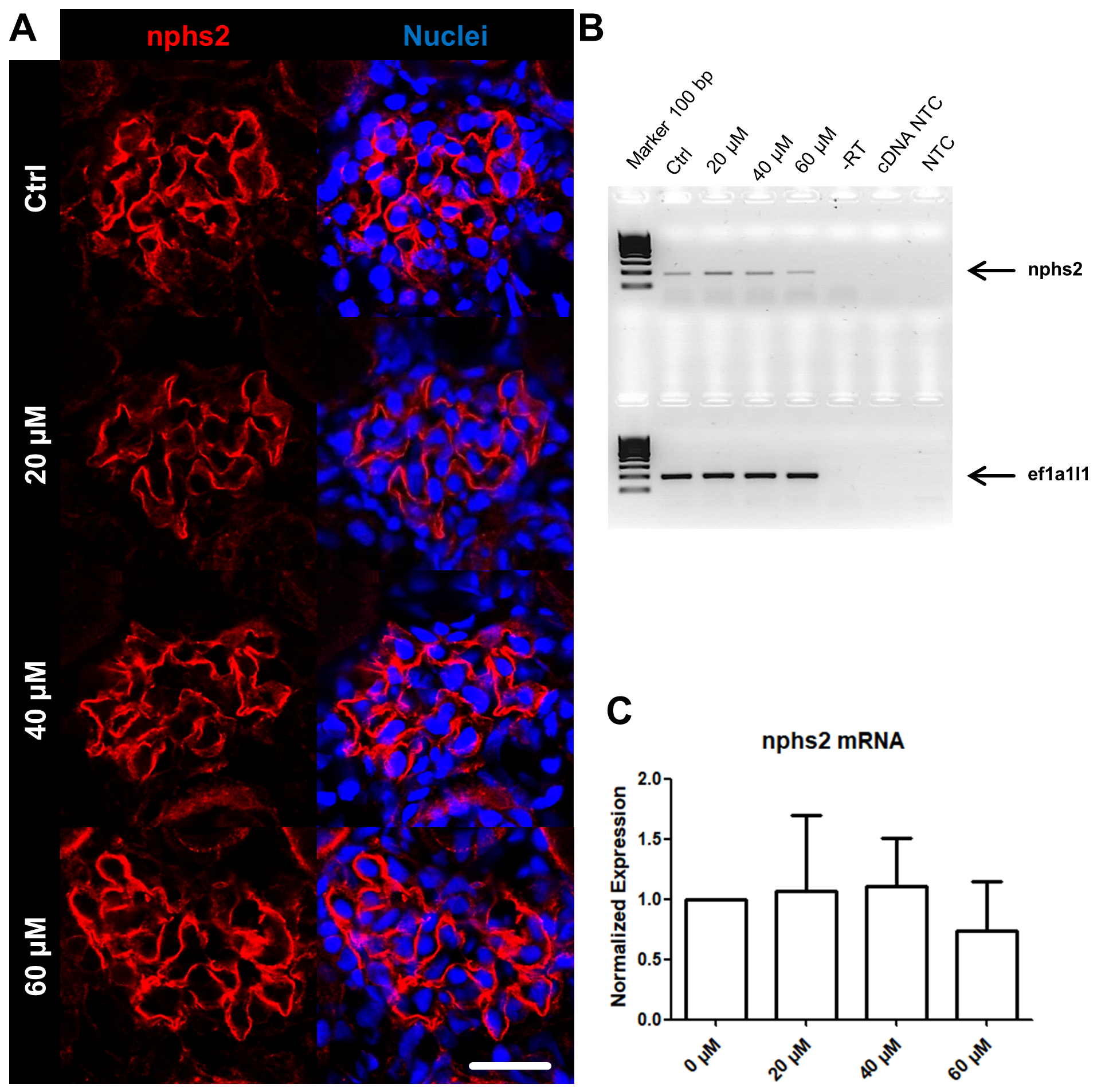

Supplement: S1 Fig — Similar to nephrin, podocin showed a meandering expression along the slit membrane in glomeruli throughout all ADR concentrations (A). These results were corroborated by podocin mRNA analysis of treated larvae via RT-PCR and RT-qPCR (B, C). Scale bar represents 10 μm. (TIF) [file pone.0242436.s001.tif]
