# Supplementary material for: Adriamycin does not damage podocytes of zebrafish larvae
Source: PLoS One. 2020 Nov 13;15(11):e0242436. doi: 10.1371/journal.pone.0242436 (PMC7665694; doi:10.1371/journal.pone.0242436)
Supplement: S1 Raw images — (PDF) [file pone.0242436.s003.pdf]

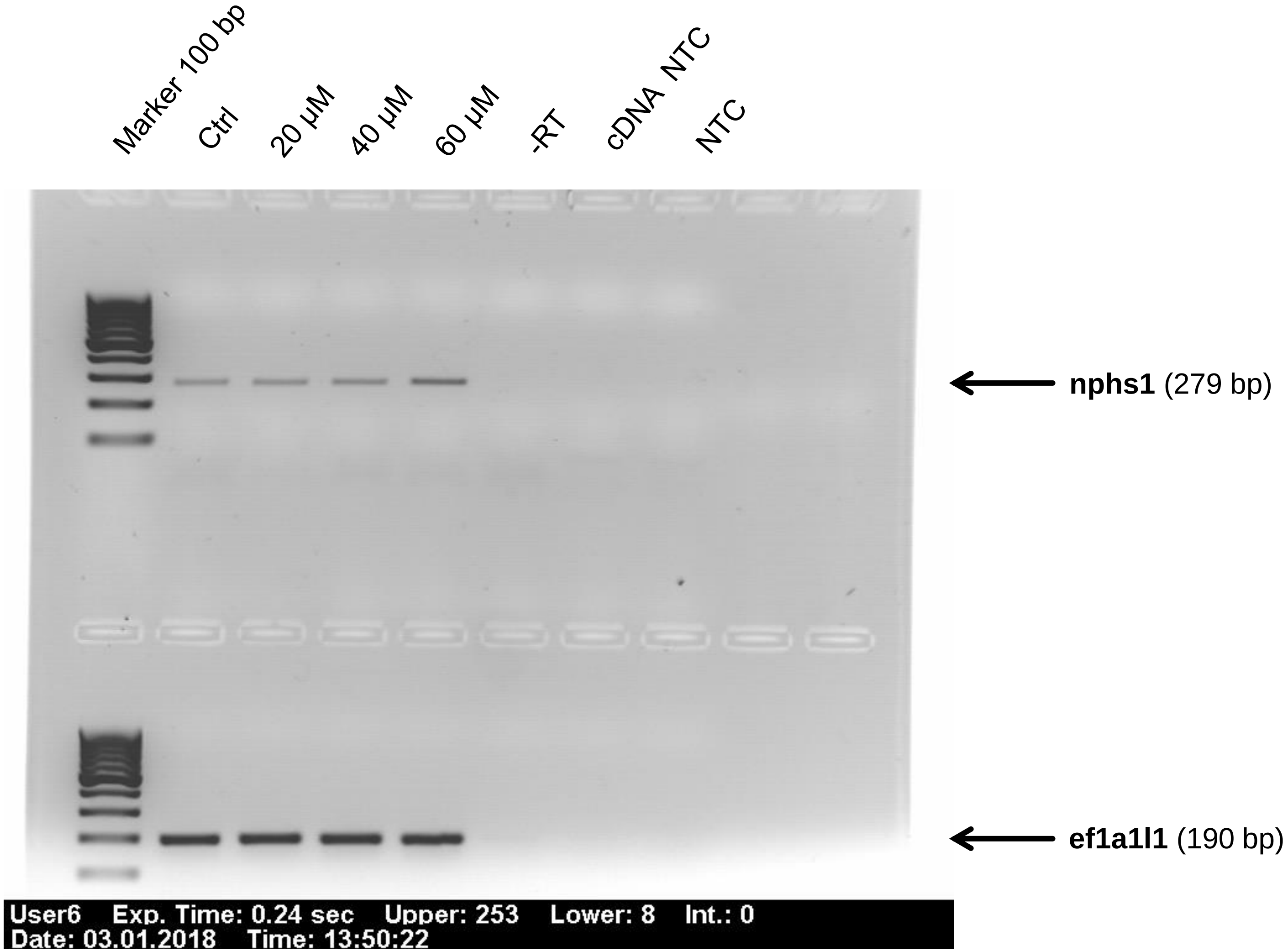

Raw gel image from Fig 3(C). Ctrl: Untreated larvae, 20-60 µM: ADR treated larvae, -RT: cDNA synthesis sample without reverse transcriptase, cDNA NTC: cDNA synthesis sample without RNA, NTC: RT-PCR sample without cDNA input. Cycle count was 31 with 10 µl sample loading volume and 5 µl marker loading volume.

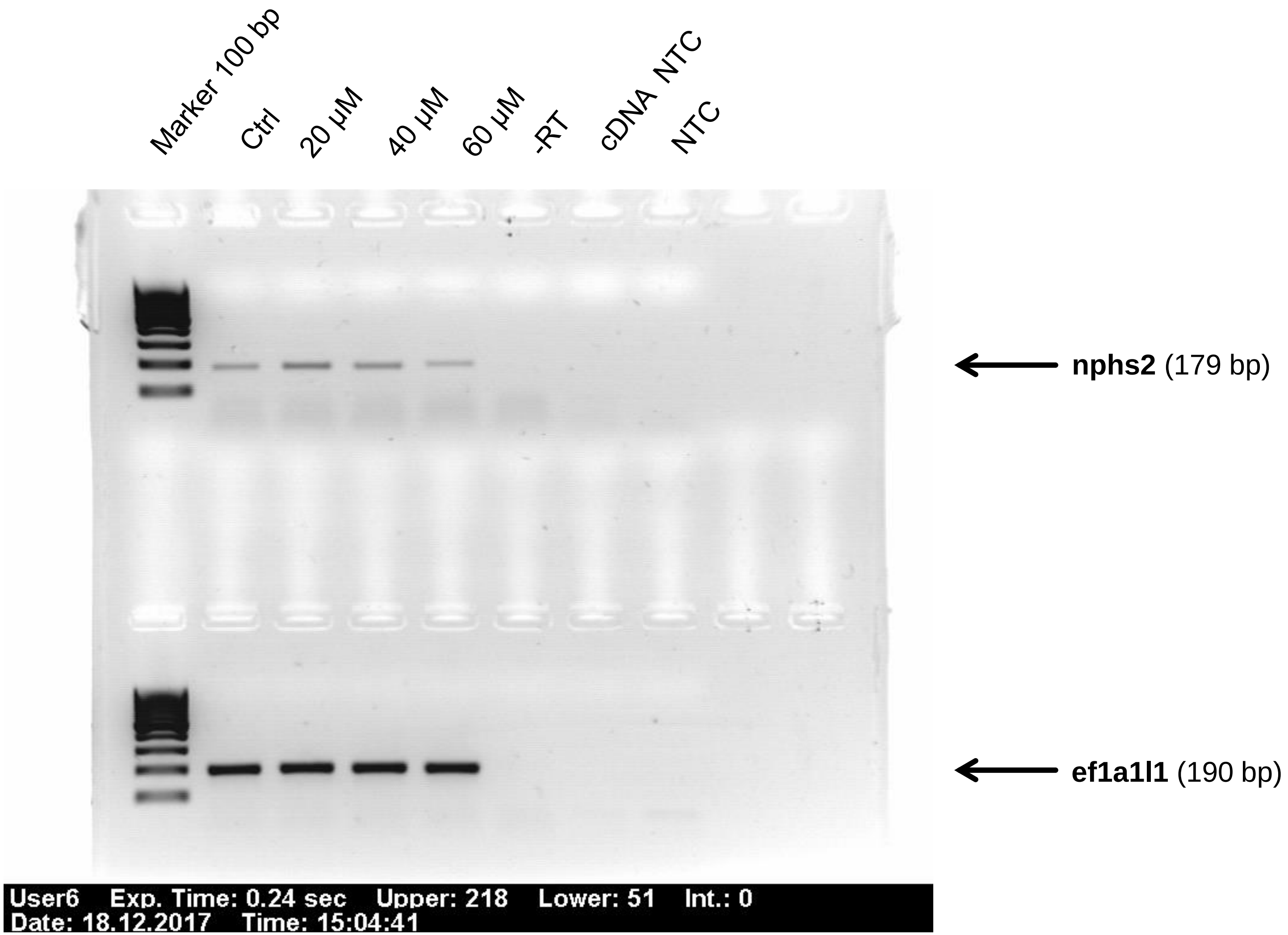

Raw gel image from S1_Fig(B). Ctrl: Untreated larvae, 20-60 µM: ADR treated larvae, -RT: cDNA synthesis sample without reverse transcriptase, cDNA NTC: cDNA synthesis sample without RNA, NTC: RT-PCR sample without cDNA input. Cycle count was 31 with 10 µl sample loading volume and 5 µl marker loading volume.
